# Supplementary material for: Modeling HBV transmission dynamics in Indonesia (2024–2030) using a SIVRM model: Evaluating optimal control strategies for elimination by 2030
Source: PLoS One. 2026 Feb 2;21(2):e0341120. doi: 10.1371/journal.pone.0341120 (PMC12863686; doi:10.1371/journal.pone.0341120)
Supplement: Attachment — Submitted filename: Hashem S. Arkok_Response to Reviewers.pdf [file pone.0341120.s003.pdf]

Modeling HBV transmission dynamics in Indonesia (2024–2030) using a SIVRM model: evaluating optimal control strategies for elimination by 2030
Author Responses

Reviewer Comment

The revised manuscript (R2) still contains a significant number of fundamental errors, suggesting that the revisions were not performed with sufficient care and diligence. The writing remains problematic, and although some issues like sentence completion and citation formatting wereaddressed, the core content has major flaws. These deficiencies are so substantial that they undermine the validity of the work.

Justification: The large number of persistent errors indicates a lack of scholarly rigor and attention to detail. This raises serious concerns about the overall quality and reliability of the research. The "very careless writing" observed in the revised version is unprofessional. A revised manuscript should demonstrate a clear effort to address all issues raised by reviewers, not introduce new problems through hasty and negligent revisions. Given the fundamental issues with the writing and content that remain in the second round of review, the manuscript does not meet the standards for publication in this journal.

Author Responses

We thank the reviewer for this helpful suggestion. Following this comment, we have carefully revised all figures in the manuscript. The figures have been regenerated with higher resolution, and the font sizes, legends, and axis labels have been adjusted to improve overall clarity and readability. In addition, we have carefully reread the entire manuscript and made the necessary revisions to improve clarity and consistency of the text. The revised figures and corresponding textual changes are highlighted in blue.
