# Supplementary material for: Modeling HBV transmission dynamics in Indonesia (2024–2030) using a SIVRM model: Evaluating optimal control strategies for elimination by 2030
Source: PLoS One. 2026 Feb 2;21(2):e0341120. doi: 10.1371/journal.pone.0341120 (PMC12863686; doi:10.1371/journal.pone.0341120)
Supplement: Attachment — Submitted filename: Hashem S. Arkok_Response to Reviewers.docx [file pone.0341120.s001.docx]

**Rebuttal Letter**

Dear PLOS ONE Editor Team,

We would like to express our sincere gratitude to you and the reviewers for the constructive and detailed feedback provided on our manuscript entitled *“Modeling HBV transmission dynamics in Indonesia (2024–2030) using a SIVRM model: evaluating optimal control strategies for elimination by 2030” (Manuscript ID: [ID])*. We have carefully considered all comments and substantially revised the manuscript accordingly. Below, we provide a detailed, point-by-point response to each editorial and reviewer comment.

**Journal Requirements**

**Comment 1:** *Ensure that the manuscript meets PLOS ONE's style requirements, including file naming.*
**Response:** We have revised the manuscript according to the PLOS ONE style guidelines, following the formatting templates provided in the links.

**Comment 2:** *Code sharing guidelines, all author-generated code should be made available without restrictions.*
**Response:** We have prepared the author-generated code used in our study and uploaded it through the submission link as supporting information.

**Comment 3:** *Data availability, all data underlying the findings must be freely available.*
**Response:** We acknowledge the journal’s data availability requirements. The dataset used in this study was obtained from BPJS Kesehatan and the Indonesian Ministry of Health. While the data do not contain individual patient identifiers, access is restricted by these institutions for legal reasons. Therefore, the data cannot be shared directly by the authors.

Qualified researchers may request access through BPJS Kesehatan by submitting a formal data request via their official website <https://e-ppid.bpjs-kesehatan.go.id/eppid/#/home/beranda> . Similarly, data from the Indonesian Ministry of Health can be requested through the Directorate of Immunization’s official website <https://upk.kemkes.go.id/new/home> . Access is subject to institutional review and approval in accordance with their regulations.

For the editors’ reference, we have uploaded official documents from BPJS Kesehatan and the Indonesian Ministry of Health confirming that these data are legally restricted and can only be accessed through their approval process.

In line with the journal’s policy, we respectfully request an exemption on the basis of legal restrictions.

**Comment 4:** *Remove figures from the manuscript file and upload them separately as TIFF/EPS.*
**Response:** We have removed all figures from the main manuscript file and uploaded them as separate TIFF files, each with the appropriate resolution. The figure legends remain included in the revised manuscript text.

**Response to Reviewer 1**

**1. Abstract & Introduction**

**Comment:** *Restructure the abstract to clearly present objectives, methodology, key results, and conclusions.*
**Response:** The abstract has been rewritten to follow a logical sequence: (1) objective, (2) methodology, (3) key findings, and (4) conclusion.

**Comment:** *Objective & novelty are not clearly defined.*
**Response:** We revised the introduction to explicitly state the primary aim and the novelty of our study **(Revised on lines 143-153)**.

**Comment:** *Add a subsection on Research Gap.*
**Response:** A new subsection entitled “Research Gap” has been added to the introduction, highlighting how this study addresses limitations in the existing literature **(Revised on lines 155-173).**

**2. Citations & Referencing**

**Comment:** *Incorrect citation format (“(1)” instead of “[1]”).*
**Response:** All citations have been reformatted to comply with the journal’s required style.

**Comment:** *Verify that all references, figures, and tables are properly cited in the text.*
**Response:** We have carefully checked and ensured that all references, figures, and tables are properly cited.

**3. Grammar & Punctuation**

**Comment:** *Numerous grammatical and punctuation errors throughout.*
**Response:** We thoroughly revised the manuscript for grammar and punctuation. All in-text citation placements have been corrected, and sentences were restructured where needed for clarity.

**4. Clarity & Redundancy**

**Comment:** *Clarify and avoid repetition in the SIVRM model statement.*
**Response:** The statement has been revised for clarity. After the first definition, only the acronym “SIVRM” is used.

**Comment:** *HBV term is overused.*
**Response:** We replaced some instances of “HBV” with synonyms such as “hepatitis B virus” or “the virus” to improve readability.

**5. Manuscript Structure**

**Comment:** *Add a paragraph at the end of the introduction summarizing the organization of the paper.*
**Response:** We have added the suggested paragraph at the end of the introduction to outline the paper's structure **(Revised on lines 174-176)**.

**6. Mathematical Presentation**

**Comment:** *Symbols must be formatted properly (e.g., β instead of “beta”).*
**Response:** All symbols in the tables have been reformatted using standard mathematical notation.

**Comment:** *Parameter estimation methods are missing.*
**Response:** We added a detailed description of parameter estimation, including fitting techniques **(Revised on lines 322-332)**.

**Comment:** *Jacobian and transmission matrices contain inaccuracies.*
**Response:** We thank the reviewer for pointing this out. We have carefully recalculated both the Jacobian and the transmission matrices, and revisions have been made where necessary. The corrected forms are now presented in the **Mathematical Analysis** section of the revised manuscript.

**Comment:** *Endemic Equilibrium (EE) and Reproduction Number (R₀) analysis is incomplete.*
**Response:** We appreciate the reviewer’s comment. In the revised manuscript, we have provided a complete and detailed calculation of the basic reproduction number, R_0_ . For the endemic equilibrium (EE), an analytical characterization is not feasible due to the high complexity of the resulting equations. Although it is possible to approximate the EE numerically, this lies beyond the primary focus of our current work. We highlight this as an interesting direction for future research, where a more thorough numerical investigation of the EE could complement our present analysis and provide additional insights. We add our future research possibilities in the final section of our paper **(lines 819 – 827).**

**7. Computational Results**

**Comment:** *Numerical results are not supported by theoretical analysis.*
**Response:** We thank the reviewer for the comment. In the revised manuscript, we strengthened the justification of our computational findings by explicitly linking them with established theoretical results. We also added a rigorous reference to support this theoretical foundation. **(Revised on 517 – 521) and (594 – 600).**

**Reviewer’s Remark on Suggested Readings**

**Response:** We thank the reviewer for recommending relevant studies. We have reviewed these papers.

**Response to Reviewer 2**

**Comment 1:** The *Similarity index is very high; please reduce it.*
**Response:** We thoroughly revised the text to improve originality and reduce the similarity index.

**Comment 2:** *Add the main findings and objective in the abstract.*
**Response:** As noted in the response to Reviewer 1, the abstract has been revised to clearly present objectives, methodology, findings, and conclusions.

**Comment 3:** *What are the benchmark cases in your study?*
**Response:** We have added explanations of benchmark cases considered in the study **(Revised on lines 528 – 531)**.

**Comment 4:**  *What are the special cases of your study?*
**Response:** We have added explanations of special cases considered in the study **(Revised on lines 601 – 604))**.

**Comment 5:** *Table needs to be referenced.*
**Response:** All tables are now explicitly referenced in the text.

**Comment 6:** *Main equations and propositions need to be referenced.*
**Response:** Equations and propositions are now properly referenced and numbered in the text.

**Comment 7:** *Punctuation is missing after some equations.*
**Response:** We corrected punctuation issues following the equations throughout the manuscript.

**Comment 8:** *Show more of the physical situation in the results and discussion.*
**Response:** We revised the results and discussion to provide more interpretation of the physical and epidemiological implications of the findings **(Added on lines 623-628 and 738-749).**

**Comment 9:** *Enhance the introduction with new publications and replace some old references.*
**Response:** We thank the reviewer for the suggestion to enhance the introduction with new publications and replace some older references. While many of the cited works remain foundational, we have added a recent reference in the vaccine paragraph (Introduction, lines 87,88) to strengthen the discussion on the challenges of HBV control and the role of vaccination.

We greatly appreciate the time and effort invested by the reviewers and editor. Their insightful comments have helped us substantially improve the clarity, rigor, and presentation of our work. We believe the revised manuscript is now significantly strengthened and meets the standards of *PLOS ONE*.

We look forward to your favourable consideration.

Sincerely,
Hashem S. Arkok

On behalf of all co-authors
