# Supplementary material for: Modeling HBV transmission dynamics in Indonesia (2024–2030) using a SIVRM model: Evaluating optimal control strategies for elimination by 2030
Source: PLoS One. 2026 Feb 2;21(2):e0341120. doi: 10.1371/journal.pone.0341120 (PMC12863686; doi:10.1371/journal.pone.0341120)
Supplement: Attachment — Submitted filename: Hashem_S._Arkok_Response_to_Reviewers_auresp_2.docx [file pone.0341120.s002.docx]

Dear PLOS ONE Editor Team,

We would like to express our sincere gratitude to you and the reviewers for the constructive feedback on our manuscript entitled “Modeling HBV transmission dynamics in Indonesia (2024–2030) using a SIVRM model: evaluating optimal control strategies for elimination by 2030”. We have carefully considered all comments and substantially revised the manuscript accordingly. Below, we provide a detailed, point-by-point response.

**Editorial & Reviewer Comments on Figures**

**Comment (Editor and Reviewer #1):** Please redraw all the figures once again. None of the figures are readable, neither the legend, nor axis level, and the figures breaks the originality.

**Response:** We sincerely thank both the editor and Reviewer #1 for highlighting this important issue. In response, we have completely redrawn and reformatted all figures to ensure clarity, readability, and compliance with PLOS ONE figure preparation guidelines. Specifically:

- All figures were regenerated in high resolution (300 and 600 dpi).
- Font sizes for legends and axis labels were improved for better visibility.
- Line thickness and color contrast were adjusted to enhance clarity and accessibility.
- Figures were validated using the PACE tool to confirm compliance with PLOS ONE requirements.

Additionally, for Figures 3 and 4, we enhanced the visualizations by refining the original code in MATLAB to redraw the figures more clearly. These adjustments do not alter the results but ensure that the presentation is more readable and consistent with the journal’s standards. We have also resubmitted the adjusted Supporting Information (S2 and S3) to ensure reproducibility of the improved figures. We believe these improvements address all concerns regarding figure readability and presentation.

**Data Availability Statement**

We have carefully reviewed our Data Availability Statement. To ensure full compliance with PLOS ONE policy, we have updated it in the revised manuscript. Specifically, we now state that:

- **All relevant data are within the manuscript and its Supporting Information files.**
- The dataset used in this study was obtained from BPJS Kesehatan and the Indonesian Ministry of Health. While the data do not contain individual patient identifiers, access is legally restricted by these institutions.
- Qualified researchers may request access through BPJS Kesehatan by submitting a formal request via their official website (https://e-ppid.bpjs-kesehatan.go.id/eppid/#/home/beranda). Similarly, data from the Indonesian Ministry of Health can be requested through the official website (https://www.badankebijakan.kemkes.go.id/en/layanan-permintaan-data/?utm_source=chatgpt.com). Access is subject to institutional review and approval. **(Revised on lines 284 – 289)**

**Response to Reviewer #2**

**Comment:** The authors has addressed all the comments satisfactorily; the paper can be accepted in the present form.

**Response:** We thank the reviewer for the positive assessment and encouraging remarks.

We greatly appreciate the time and effort invested by the reviewers and the editor. Their insightful comments have helped us substantially improve the clarity, presentation, and technical quality of our work. We believe the revised manuscript is now significantly strengthened and meets the standards of PLOS ONE.

We look forward to your favorable consideration.

Sincerely,
Hashem S. Arkok

On behalf of all co-authors
